# ΔNp63α facilitates proliferation and migration, and modulates the chromatin landscape in intrahepatic cholangiocarcinoma cells

**DOI:** 10.1038/s41419-023-06309-7

**Published:** 2023-11-27

**Authors:** Anghui Peng, Xiaowen Lin, Quanli Yang, Yihao Sun, Ruiyan Chen, Bing Liu, Xinyang Yu

**Affiliations:** 1 Guangdong Provincial Key Laboratory of Tumor Interventional Diagnosis and Treatment, Zhuhai Institute of Translational Medicine, Zhuhai People’s Hospital Affiliated with Jinan University, Jinan University, Zhuhai, China; 2 Zhuhai Interventional Medical Center, Zhuhai Precision Medical Center, Zhuhai People’s Hospital, Zhuhai Hospital Affiliated with Jinan University, Jinan University, Zhuhai, China; 3 Department of Dermatology, Zhuhai People’s Hospital Affiliated with Jinan University, Jinan University, Zhuhai, China

**Keywords:** Bile duct cancer, Cancer genomics

## Abstract

p63 plays a crucial role in epithelia-originating tumours; however, its role in intrahepatic cholangiocarcinoma (iCCA) has not been completely explored. Our study revealed the oncogenic properties of p63 in iCCA and identified the major expressed isoform as ΔNp63α. We collected iCCA clinical data from The Cancer Genome Atlas database and analyzed p63 expression in iCCA tissue samples. We further established genetically modified iCCA cell lines in which p63 was overexpressed or knocked down to study the protein function/function of p63 in iCCA. We found that cells overexpressing p63, but not p63 knockdown counterparts, displayed increased proliferation, migration, and invasion. Transcriptome analysis showed that p63 altered the iCCA transcriptome, particularly by affecting cell adhesion-related genes. Moreover, chromatin accessibility decreased at p63 target sites when p63 binding was lost and increased when p63 binding was gained. The majority of the p63 bound sites were located in the distal intergenic regions and showed strong enhancer marks; however, active histone modifications around the Transcription Start Site changed as p63 expression changed. We also detected an interaction between p63 and the chromatin structural protein YY1. Taken together, our results suggest an oncogenic role for p63 in iCCA.

## Introduction

Cholangiocarcinoma (CCA), including intrahepatic CCA (iCCA), perihilar CCA, and distal CCA, accounts for 3% of all gastrointestinal tumours [1]. These subtypes differ in their epidemiology, origin, etiology, pathogenesis, and treatment [2]. iCCA, a rare but highly lethal hepatobiliary disease, is the second most common primary hepatic malignancy [3]. iCCA arises from the epithelial cells of the intrahepatic small bile ducts [3]. Its development is correlated with liver cirrhosis, intrahepatic bile duct stones, and hepatitis virus infection. At the time of iCCA diagnosis, a large percentage of patients have reached an advanced pathological stage and show poor responsiveness to existing therapies [4]. Researchers have attempted to understand the complexity of iCCA; however, the epigenetic mechanisms underlying iCCA have not yet been fully elucidated.

The epigenetic dysregulation of gene expression plays a vital role in tumourigenesis. With the rapid development of next-generation sequencing technology, increasing evidence has shown that the accumulation of epigenetic alterations in cells can lead to abnormal gene expression, such as the activation of oncogenes or the inactivation of tumour suppressor genes, which in turn regulate tumour progression [5]. Epigenetic modifications occur during the early stages of tumour development [6]. The interaction of transcription factors with target gene promoters, enhancers, and other regulatory elements is a prerequisite for regulating gene expression. However, in eukaryotes, nucleosomes function as the fundamental building blocks of chromatin and prevent transcription factors from directly accessing the target DNA. Chromatin remodelling is required to help expose sequences wrapped inside nucleosomes so that transcription factors and other cofactors can be recruited and ultimately regulate target gene expression [7]. Pioneer transcription factors are transcription factors that can directly bind to nucleosomal DNA to enable gene expression in closed chromatin [8]. Pioneer transcription factors either work independently or cooperate with other chromatin remodellers to open inaccessible chromatin regions [9]. Pioneer transcription factor-mediated chromatin remodelling is an essential step in activating tumour-related gene expression [10]. Well-studied pioneer factors such as p53 and FOXA1 have been demonstrated to not only play an important role in cellular processes but also in cancer development [11].

p63 is a member of the p53/p63/p73 family and our previous study has shown that it also functions as a pioneer factor [12]. p63 is consistently expressed in basal cells of the epidermis and is a key mediator in the establishment and maintenance of epithelial identity [13]. Its expression has also been detected in squamous cell carcinoma of the lung [14], colorectal cancer [15], skin squamous cell carcinoma and other skin tumours including Bowen’s disease, actinic keratosis and seborrhoeic keratosis [16]. Buck et al. [17] demonstrated that in keratinocytes, p63-bound sites were enriched with active histone modifications but lacked repressive epigenetic signatures. The analysis of p63 targets has revealed a preference for enhancer and super-enhancer sites [18]. Recent studies have shown that p63 mediates the establishment of epithelial enhancers; p63-bound enhancers display increased activity, whereas the loss of p63 impairs enhancer activity and affects target gene expression [19].

In mammals, the p63 gene is alternatively spliced to produce multiple isoforms that can be divided into two major groups: TAp63, which contains an N-terminal transcriptional activation domain, and ΔNp63 lacking a transactivation domain [20]. Early studies have suggested that ΔNp63 is not involved in transcriptional activation and is a repressive protein [13]; it is now believed that ΔNp63 can activate or inhibit gene expression by recruiting cofactors [21]. ΔNp63α is the most predominant p63 isoform in epithelial cells and possesses the majority of p63 biological functions [22]; dysregulation of ΔNp63α promotes the development of skin squamous carcinoma [23], triple-negative breast cancer [24], and oral cancer [25].

To date, p63 expression has not been detected in normal human bile duct epithelial cells, but Steurer et al. [26, 27] detected abnormal overexpression of p63 in a group of iCCA tissue samples. Interestingly, ectopic expression of p63 in the mouse bile duct epithelium contributes to iCCA [28]. However, the role of p63 in iCCA has not yet been fully elucidated. Here, we explored how p63 affects the gene expression profiles in iCCA and sought to understand how it regulates gene expression, as well as chromatin landscape alteration, to further understand the epigenetic regulatory role of p63 in iCCA. Our study investigated the role of p63 in iCCA and provided new insights into its complexity.

## Materials and methods

### Cell culture

The intrahepatic CCA cell lines RBE, CCLP1, HUH28, HCCC-9810, and HuCC-T1 were cultured in RPMI-1640 medium (Gibco) supplemented with 10% FBS (Gibco) and 100 U/mL penicillin/streptomycin (Gibco). Normal intrahepatic biliary epithelial cells (HIBEpiCs) were cultured in HIBEpiC-specific complete medium (Procell Co., Ltd.) supplemented with 10% FBS (Gibco). The extrahepatic CCA cell line TFK-1 was cultured in Dulbecco’s modified Eagle’s medium (DMEM; Gibco) supplemented with 10% FBS (Gibco). Cells were cultured at 37 °C under 5% CO_2_ and 95% humidity conditions. The medium was refreshed every alternate day. A MycoBlue Mycoplasma Detector (Vazyme) was used before utilizing the cells, and no mycoplasma contamination was observed during cell culturing.

### Lentivirus and retrovirus infection

To construct a p63-overexpressing cell line, a pEZ-Lv201 plasmid containing the ΔNp63α coding sequence was constructed; the p63-overexpressing plasmid was then transfected into RBE cells. Cells transfected with an empty vector served as negative controls. A psi-LVRU6MP plasmid containing shRNA targeting ΔNp63α was transfected into CCLP1 cells. Cells transfected with scrambled control shRNA were utilized as negative control cells. All transfections were performed using the Lenti-Easy Packaging System kit (Genechem, LPK001), following the manufacturer’s instructions.

### Quantitative reverse transcription real-time PCR

Total RNA was extracted from the cells using TRIzol (Invitrogen), and cDNA was generated using the HiScript 1st Strand cDNA Synthesis Kit (Vazyme, R111-01). Quantitative reverse transcription real-time PCR (qRT-PCR) was performed using SYBR® Premix Ex Taq™II (TAKARA) on a 7300 Real-Time Fluorescence PCR Detection System (ABI). RNA expression was normalized by GAPDH expression level and quantified using the 2^-ΔΔCt^ method. The primers used for qRT-PCR are listed in Table S1.

### Western blot

Cells were harvested and lysed in RIPA buffer (Thermo Fisher Scientific) supplemented with protease inhibitors (Thermo Fisher Scientific). After centrifugation, the supernatant was collected, and the protein concentration of each sample was determined using a BCA kit (Thermo Fisher Scientific). Protein samples were loaded into SDS-PAGE gel, electrophoresed, and transferred to a PVDF membrane, followed by incubation with blocking buffer (5% BSA). The membrane was probed with primary antibodies at 4 °C overnight, followed by incubation with secondary antibodies at room temperature. Immunoblots were visualized using an imaging system (Omega LumC) with an Immobilon western chemiluminescent horseradish peroxidase (HRP) substrate (Millipore). The primary antibodies used were anti-p63 (ab124762; Abcam) and anti-actin (KC-5A08; PT Solution Biotechnology). The secondary antibodies we applied included goat anti-rabbit IgG H&L (HRP) (ab6721; Abcam) and goat anti-mouse IgG H&L (HRP) (ab6789; Abcam). Full and uncropped western blots are available in Figure S10.

### Co-immunoprecipitation

Whole cell lysates for immunoprecipitation were prepared using RIPA lysis buffer (87787, Thermo Fisher Scientific) supplemented with protease inhibitors. Cell lysates were then incubated with anti-ΔNp63 (67825 S; Cell Signaling Technology), anti-YY1 (sc-7341X), or IgG (normal mouse IgG, sc-2025; SantaCruz, and normal rabbit IgG, NI01; Sigma-Aldrich) antibodies overnight at 4 °C with shaking. The immune complex solution was incubated with protein A/G magnetic beads (Beyotime Biotechnology) for 1 h at room temperature and washed six times with RIPA lysis buffer to remove unbound immune complexes. The bound immune complexes were dissociated from the beads using 2×SDS-PAGE loading buffer (P0015B; Beyotime) for western blot analysis. Full and uncropped western blots are available in Figure S10.

### Cell proliferation assay

Cells were plated in a 96-well culture plate at 1000 cells/well in six replicate wells, and the total cell numbers in each well were counted at 24 h, 48 h, and 72 h of incubation using a Cell Counting Kit-8 (Beyotime Biotechnology) according to the manufacturer’s instructions. The absorbance was measured at 450 nm. Each experiment was performed in sextuplicate.

### Wound healing assay

Wound healing assays were performed to investigate the migration ability of cells in vitro. Cells were plated in a 6-well culture plate at 2 × 10^6^ cells/well. Scratch wounds were created using a sterilized pipette tip. Photographs were taken at 0 h, 24 h, and 48 h. The width of the wounds was measured using ImageJ software to calculate the wound healing percentage. Each experiment was performed in triplicate.

### Transwell assay

Cell invasion was determined using the Matrigel Transwell invasion assay. The porous membranes (pore size 3·0 um) were precoated with Matrigel (Corning), and the cell culture medium supplemented with 10% FBS was added to the lower chamber. Cells (10^5^ cells/mL) were resuspended in serum-free medium and 200 μL were added to the upper chambers. After 48 h of incubation, the invading cells that migrated to the lower side of the membrane were fixed, stained, and counted.

### Colony formation assay

Cells (1000 cells/well) were inoculated into 6-well plates in triplicate. The culture medium was changed every 2 days. After 14 days of incubation, when macroscopic apophyses were observed, the old culture medium was removed, and the cells were gently rinsed with PBS. Cells were then fixed with 4% paraformaldehyde (Biotechnology) for 15 min and stained with crystal violet (Biotechnology) for 20 min. The number of colonies was measured using ImageJ software.

### Immunohistochemistry

Immunohistochemistry (IHC) was performed on paraffin-embedded tissue sections of human intrahepatic CCA samples as previously described [29]. Briefly, paraffin sections were deparaffinized for 2 h at 60 °C, followed by rehydration using an alcohol series and sodium citrate buffer. The sections were treated with 3% hydrogen peroxide and blocked with 5% normal goat or mouse serum. Sections were incubated with anti-p63 antibody (ab124762; Abcam) at 4 °C overnight. Immunohistochemical staining was performed using HRP conjugates and diaminobenzidine. The images were captured using a confocal microscope (Olympus). The IHC score was computed by multiplying the staining intensity grade (grades of 0, 1, 2, and 3 implied negative, weak-positive, moderate-positive, and strong-positive, respectively) by the positive rate score (scores of 0, 1, 2, 3, and 4 represented positive areas of 0, 1–25%, 26–50%, 51–75%, and 76–100%, respectively). Two proficient pathologists independently assessed the scores. Tissue microarrays of human intrahepatic CCA tissues (HIBDA160PG01) were purchased from Shanghai Outdo Biotech Company (Shanghai, China). The study was approved by the Ethics Committee of Shanghai Outdo Biotech Company.

### Survival analysis

Public RNA-seq data and clinical data of patients with CCA were downloaded from The Cancer Genome Atlas (https://portal.gdc.cancer.gov/) [30] or from The National Omics Data Encyclopedia (NODE) database (https://www.biosino. org/node/project/detail/OEP001105) [31]. Intrahepatic CCA cases were selected and divided into two groups according to the mean expression value of p63: the p63-high expression group and the p63-low expression group. The clinical information, including age, sex, race, origin site, tumour state, and outcome, is displayed in Table S2 or can be found in the reference [31]. Survival analysis was performed using the Kaplan–Meier method with the log-rank test to analyse the difference between groups; *p* < 0.05 was considered statistically significant. Statistical analysis was performed using the R package “survival”.

### Chromatin immunoprecipitation and data analysis

Cell pellets were collected and washed with PBS for each chromatin immunoprecipitation (ChIP-seq) experiment. Chromatin cross-linking, isolation, sonication, and immunoprecipitation were performed as previously described [12]. Sheared chromatin from 1 million cells was used for each ChIP-seq experiment for H3K27ac, H3K27me3, H3K4me1, and H3K4me3, using the iDeal ChIP-seq kit for histones (Diagenode: C01010051). Sheared chromatin from 4 million cells was used for each ChIP-seq experiment for ΔNp63α and YY1 using the iDeal ChIP-seq kit for TFs (Diagenode: C01010055). ChIP for ΔNp63α was carried out using 5 μL of anti-ΔNp63 (67825 S; Cell Signaling Technology) antibody per IP. ChIP for YY1 was carried out using ~6 μg of anti-YY1 (sc-7341X; Santa Cruz) antibody. ChIP for histone marks H3K27ac, H3K27me3, H3K4me1, and H3K4me3 was performed using 3 μg each of H3K27ac (ab4729; Abcam), H3K27me3 (ab6002; Abcam), H3K4me1 (ab8895; Abcam), and H3K4me3 (04-745; Merck Millipore) antibody, respectively. Sequencing libraries were prepared using a TruePrep DNA Library Prep Kit V2 for Illumina (Vazyme). Samples were sequenced on NovaSeq using 150-bp paired-end sequencing. Raw sequencing reads were analysed using a previously published workflow [32]. The sequenced reads were mapped using Bowtie2 [33]. Fully processed and filtered BAM files were merged to represent the union of all available replicates for further analysis. MACS2 was used to perform peak-calling [34]. Quality tests and visualizations were conducted using R and IGV.

### RNA-seq and data analysis

Total RNA was extracted and assessed using an RNA Nano 6000 Assay Kit on a Bioanalyzer 2100 system (Agilent Technologies). mRNA was purified from the total RNA using poly-T oligo-attached magnetic beads. The enriched and purified mRNA was subsequently used to generate a sequencing library, which was sequenced on an Illumina NovaSeq 6000. The end reading of the 150-bp pairing was generated. After sequencing, Fastq files were processed using TrimGalore to remove adaptor sequences, and the trimmed files were aligned to the human genome (hg38) using Hisat2. Feature counts were used to determine the number of reads mapped to each gene. Each RNA-seq experiment was performed in triplicate. Differential expression analysis was performed using the R package DESeq2. *P*-values were adjusted using the Benjamini–Hochberg approach to control the false discovery rate (FDR). Adjusted *p*-value < 0.05 and |log2(fold change)| > log2(1.2) were set as the thresholds for significant differential expression, unless otherwise specified. Functional enrichment analysis was conducted using the R package “clusterProfiler”.

### Transposase-accessible chromatin assay

Transposase-accessible chromatin assay (ATAC-seq) was performed as previously reported [35]. For each ATAC-seq assay, 50,000 cells were collected and washed with PBS. Cells were lysed in 50 μL cold lysis buffer (10 mM Tris-HCl, pH 7.4, 10 mM NaCl, 3 mM MgCl2, and 0.1% IGEPAL CA-630). After discarding the supernatant, cells were used for the transposition reaction. The transposition reaction mixture (25 μL 2× TD buffer, 2.5 μL Tn5 transposase, 22.5 μL nuclease free H_2_O) was added to the cell pellet for transposase fragmentation, and the mixture was incubated at 37 °C for 30 min. Then, DNA purification was performed using a MinElute PCR purification kit (QIAGEN). The transposed DNA was eluted in 10 μL elution buffer (10 mM Tris buffer, pH 8). Library construction was followed by: 10 μL transposed DNA, 10 μL nuclease free H_2_O, 2·5 μL of 25 μM custom Nextera PCR primer 1, 2.5 μL of 25 μM custom Nextera PCR primer 2 (contains barcode), and 25 μL NEBNext high-fidelity 2x PCR Master Mix; 1 cycle of 72 °C for 5 min, 98 °C for 30 s, 9 cycles of 98 °C for 10 s, 63 °C for 30 s, and 72 °C for 1 min. The library was purified and its quality was determined using an Agilent High Sensitive DNA Kit and bioanalyzer. ATAC-seq libraries were sequenced with an Illumina NovaSeq using 150-bp paired-end sequencing in duplicate. Trimming, alignment, and peak calling were performed, and the biological replicates were merged for further analysis.

### Statistics

The experiments were repeated at least three times. Differences were compared using Student’s *t*-test, and a *p*-value less than 0.05, unless otherwise specified, was considered statistically significant.

## Results

### Correlation of p63 expression with outcomes in patients with iCCA

**Fig. 1:**
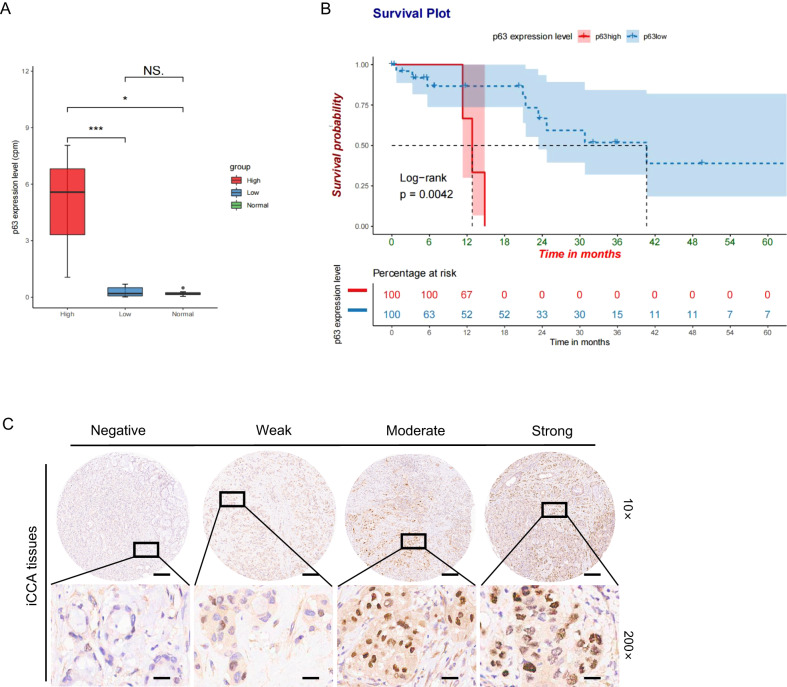
p63 expression in iCCA and association with survival. **A** p63 expression level in the p63-high group was significantly different from those in the p63-low and normal groups, while it was not significantly different between the p63-low and normal groups. *P*-value was calculated using a *t*-test. **B** Kaplan–Meier analysis comparing the survival of iCCA patients with low p63 expression to patients with high p63 expression using the mean value as the threshold. *P*-value was calculated using the log-rank test. **C** Representative IHC images of p63 protein expression in iCCA tissue samples.

UALCAN [36] analysis showed limited expression of p63 in the normal people group compared with p63 expression in the CCA patient group (Figure S1A); such results were in agreement with previous reports [26, 27]. To assess the prognostic value of p63 expression profiles in iCCA, we downloaded the RNA-seq data from The Cancer Genome Atlas (TCGA)-Cholangiocarcinoma [37] and selected the iCCA samples to perform survival analysis: we defined patients with p63 expression levels higher than the mean value as the p63-high group, while the others were the p63-low group. p63 expression in the p63-high group was significantly different from that in the p63-low and non-tumour/normal groups (Fig. 1A). The p63-high group displayed significantly poor survival (*p* = 0.0042, Fig. 1B), indicating an association between p63 expression and patient outcomes. The p63 levels in each patient are shown in the Supplementary Data (Figure S1B). We also attempted to classify iCCA patients into p63-high, medium, and low groups according to the upper, medium, and lower quartile of p63 expression levels, respectively, and obtained similar results (Figure S1C). We next examined other clinical elements, such as age and sex, but none showed any significant correlation with iCCA patient survival rates (Figure S1D, E). Hazard ratio analysis further revealed that tumour stage (HR > 10) and p63 (HR > 2) significantly increased the hazard of iCCA outcome, but not other elements such as race, age, sex, or housekeeping gene Actb (Figure S1F). We repeated survival analysis in a much larger dataset of 255 treatment-naive iCCA patients from The National Omics Data Encyclopedia (NODE) database (OEP001105) [31], and also detected a significant correlation between p63 expression and patient survival (Figure S2), consistent with what we observed in the TCGA. Moreover, the expression of p63 protein was determined by IHC in tumour tissue samples from 155 iCCA patients in a tissue microarray (Table S3), nuclear staining of p63 was detected in 19 out of 155 samples with varied expression levels (Fig. 1C).

### Identification and quantification of p63 isoform expression in iCCA

**Fig. 2:**
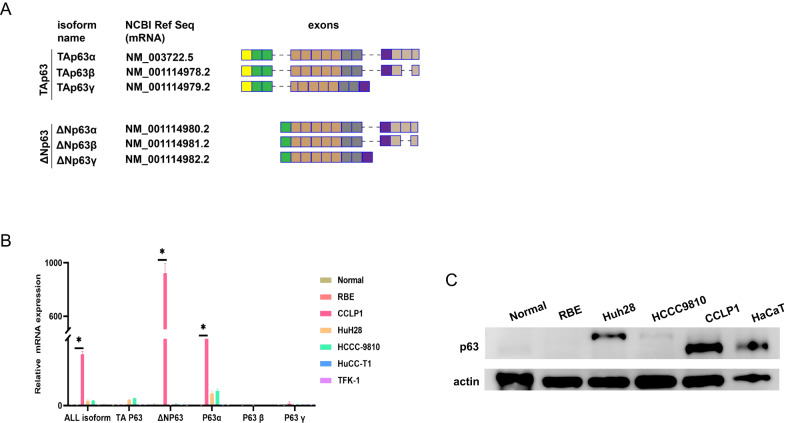
Identification and quantification of p63 isoforms expressed in iCCA. **A** Schematic illustration of different p63 isoforms. **B** qRT-PCR detected the expression of different p63 isoforms in human normal intrahepatic biliary epithelial and intra- and extrahepatic CCA cell lines. Differences were considered significant at *p* < 0.01. **C** Western blot assay confirmed the expression of p63 protein in human iCCA cell lines. HaCaT was used as the positive control, and the normal intrahepatic biliary epithelial cell line was used as the negative control.

Given the contradictory relationships between p63 isoforms and patient outcomes in the literature, we identified the major p63 isoform expressed in iCCA by analyzing different iCCA cell lines, including RBE, CCLP1, Huh28, HCCC-9810, and HuCC-T1, and also tested one extrahepatic cholangiocarcinoma cell line TFK-1; the normal immortalized human intrahepatic biliary epithelial cell line (HIBEpiC) was used as a negative control. We first analysed p63 RNA levels in all the cell lines mentioned above, and isoform-specific primers were designed to perform qPCR assays (Fig. 2A, Table S1). Only in CCLP1 was p63 expression significantly different from the normal biliary epithelial cell line (Fig. 2B), which was further confirmed as the ΔNp63α isoform (Figure S3). There appeared to be a limited TAp63α isoform expressed in Huh28 and HCCC-9810 cells (Figure S3). However, no p63 isoforms were detected in RBE and HuCC-T1 cells (Fig. 2B, Figure S3). We next performed Western blot on CCLP1, Huh28, HCCC-9810, and RBE cells, and used HaCaT, which predominantly expresses the ΔNp63α isoform, as a positive control [38]. Consistent with the qPCR results, CCLP1 displayed the strongest p63 expression compared to the other iCCA cell lines, whereas RBE did not express p63 (Fig. 2C). Our results were consistent with those of previous studies, in which p63 positivity was only detected in a portion of iCCA tissues.

### Effect of knockdown and overexpression of p63 on biological characteristics of iCCA cells

We identified the expression of ΔNp63α and TAp63α in different iCCA cell lines which has not been reported before. ΔNp63 isoforms can exert dominant-negative effects over TAp63 activities, antagonize TAp63 function in target gene regulation, and activate distinct gene targets not induced by TAp63 isoforms [39]. To determine the biological significance of p63 in iCCA development and progression, we knocked down p63 in ΔNp63α-expressing cell line (CCLP1) and TAp63α-expressing cell line (Huh28, HCCC9810) respectively, as well as overexpressing ΔNp63α in no-p63-expressing iCCA cell line (RBE).

**Fig. 3:**
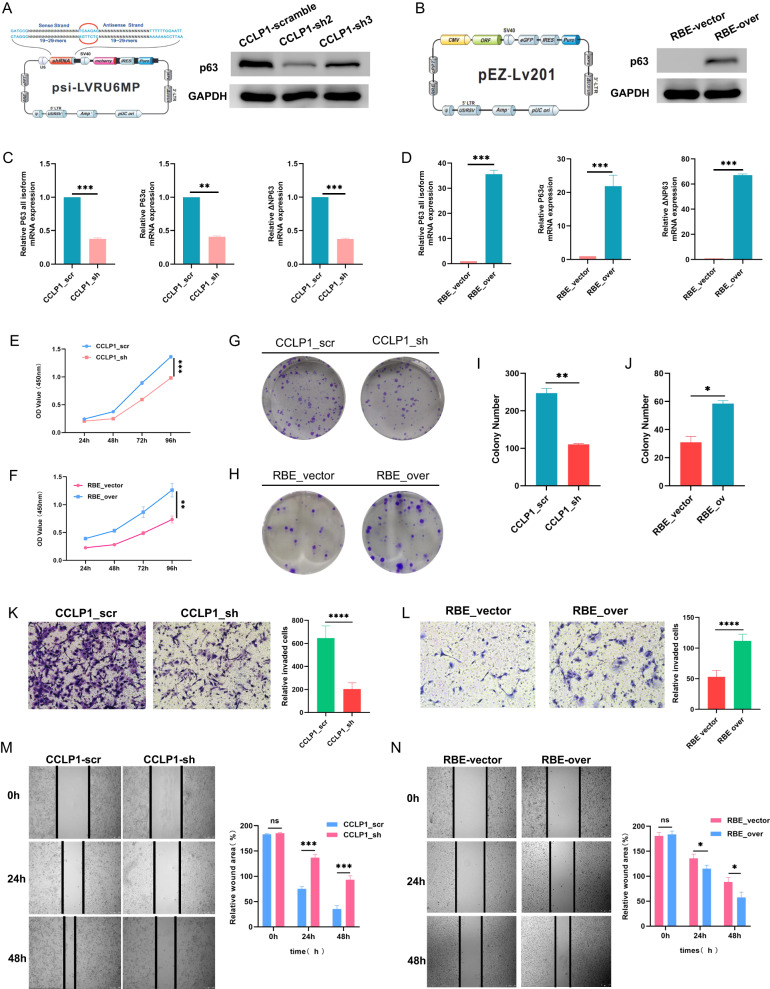
p63 expression affecting biological characteristics of iCCA cells. **A** CCLP1 cells were transfected with different shRNA-expressing plasmids against the p63 gene to generate CCLP1_sh2 and CCLP1_sh3; scrambled shRNA was used as a negative control to generate CCLP1_scr. **B** RBE cells were transfected with plasmids encoding ΔNp63α to generate RBE_over, and the empty vector-transfected cells were regarded as RBE_vector. **C** qRT-PCR was performed to test the p63 expression in CCLP1 after transfection. CCLP1_sh2 displayed high knockdown efficiency and was regarded as CCLP1_sh in subsequent assays. **D** qRT-PCR was performed to test the p63 expression in RBE after transfection. **E** CCK-8 test showed that p63 downregulation significantly impeded the proliferation of CCLP1. **F** CCK-8 test showed that p63 overexpression significantly promoted the proliferation of RBE cells. **G** Colony formation assay of CCLP1_scr and CCLP_sh. Statistical results are shown in **I**. **H** Colony formation assay of RBE_vector and RBE_over. Statistical results are shown in **J**. **K** Transwell assay revealed that the migration ability of CCLP1 was significantly decreased when p63 was knocked down. **L** Transwell assay revealed that the migration ability of RBE was significantly increased when p63 was overexpressed. Wound healing assay showed that p63 knockdown reduced the migration of CCLP1 cells **M** and p63 overexpression increased the migration of RBE cells **N**. Data shown are representative results of repeated experiments. *P*-values were calculated using *t*-tests; * indicates *p* < 0.05, ** indicates *p* < 0.01, *** indicates *p* < 0.001, and **** indicates *p* < 0.0001.

We performed p63 knockdown in CCLP1 and p63 overexpression in RBE (Fig. 3A, B). Two short hairpin RNAs (sh2 and sh3) were designed, of which sh2 showed higher p63 knockdown efficiency; therefore, we continued to use sh2 (CCLP1_sh) in the subsequent experiments (Fig. 3A). Engineered iCCA cell lines were successfully generated (Fig. 3C, D) and displayed dramatic phenotypes. The CCK-8 assay revealed that after p63 knockdown, CCLP1_sh displayed a significantly decreased proliferation rate compared with CCLP1_scr, while the ΔNp63α-overexpressing cells RBE_over proliferated much faster than the RBE_vector cells (Fig. 3E, F). Moreover, the number of colonies decreased in CCLP_sh compared to CCLP1_scr (Fig. 3G, I) and increased in RBE_over compared to RBE_vector (Fig. 3H, J). The transwell migration assay further indicated that the cell invasion capability was significantly decreased in CCLP1_sh compared to that in CCLP1_scr (Fig. 3K), but was enhanced in RBE_over compared to that in RBE_vector (Fig. 3L). Consistent results were also observed in the scratch assay, in which the cell migration ability was decreased in CCLP1_sh cells but increased in RBE_over cells (Fig. 3M, N).

We next performed p63 knockdown in Huh28 and HCCC9810, repression of TAp63α showed opposite results which distinct from ΔNp63α suppression (Figure S4), indicating that TAp63α expression does not promote cancer progression in iCCA.

These results demonstrated that ΔNp63α plays an oncogenic role in iCCA by promoting cell proliferation, migration and invasion. In the subsequent experiments, we only focused on ΔNp63α in iCCA (if not specified, p63 in the following text refers to ΔNp63α).

### Effect of abnormal p63 expression on transcriptome profiles of iCCA

**Fig. 4:**
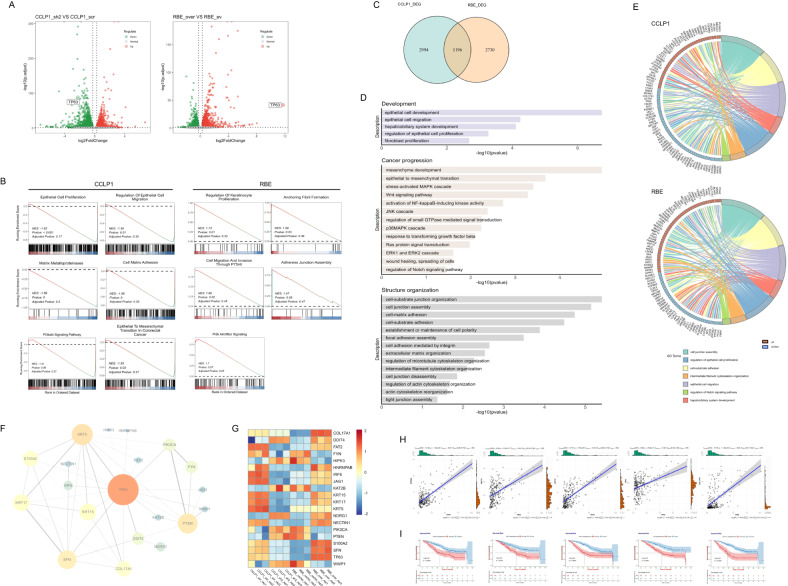
Abnormal p63 expression changes iCCA transcriptome. Significant differentially expressed genes (DEG) (|log2FC | > log2(1.2) and padj < 0.05) were identified by comparing CCLP1 (CCLP1_sh2 vs CCLP1_scr) and RBE (RBE_over vs RBE_vector) RNA-seq data. **A** Volcano plot of all detected genes using log2(FoldChange) as x-axis and-log10(p.adjust) as y-axis. Upregulated DEG were shown in red, downregulated DEG were shown in green, while not significant altered genes were shown as grey dots; the p63 gene in each group is marked with a circle. **B** Significantly enriched terms (pvalue < 0.05) generated by GSEA analysis in CCLP1 group and RBE group respectively. NES > 0 indicates activated terms, NES < 0 indicates suppressed terms. **C** Venn diagram shows the 1196 overlapped DEG from CCLP1_DEG and RBE_DEG. **D** GO analysis of overlapped DEG showed significantly enriched GO terms which were classified into three subgroups. **E** Chord diagrams display enriched GO terms and the expression pattern of associated genes. **F** PPI network with identified hub genes correlated with p63. **G** Heatmap of the hub genes expression level. **H** Correlation between p63 and hub genes was analyzed by Pearson correlation coefficient (r), *p* < 0.05 and *r* > 0.40 indicates strong positive correlation. **I** Survival analysis of hub genes in iCCA patients from NODE dataset (OEP001105), with the mean value as threshold of high-expression group and low-expression group. P-value was calculated using the log-rank test, *p* < 0.05 was considered as statistically significant.

To investigate the changes in the expression profile by overexpressing/knocking down p63, we performed RNA-seq analysis on RBE_over, RBE_vector, CCLP1_sh, and CCLP1_scr. CCLP1_sh vs. CCLP1_scr and RBE_over vs. RBE_vector were regarded as two different groups, and significant differentially expressed genes (DEG) (|log2FC | > log2(1.2) and padj < 0.05) are shown in the volcano plot (Fig. 4A); the top 100 most variable genes in these two groups were selected to draw a heatmap (Figure S5). We next picked out CCLP1_DEG and RBE_DEG and analyzed their expression pattern in the transcriptomic profiles of 16 iCCA tumour tissues and 7 non-tumour tissues from the Human Gene Expression Omnibus database (GSE32879) [40], respectively. Interestingly, these DEG, which were significantly affected by p63 expression in iCCA cell lines (either significantly elevated or significantly downregulated), had an overall upregulated expression pattern in iCCA tumour tissues compared to non-tumour counterparts (Figure S6), indicating that p63 might affect iCCA progression by orchestrating the expression of a group of essential genes during tumour development.

We next performed enrichment analysis on CCLP1_DEG and RBE_DEG respectively. Gene Set Enrichment Analysis (GSEA) found significantly suppressed terms such as epithelial cell proliferation, regulation of epithelial cell migration, matrix metalloproteinases, and cell matrix adhesion in CCLP1 group (Fig. 4B), whereas activated terms associated with cell proliferation, migration, cell junctions in RBE group (Fig. 4B), which are in accordance with the phenotypes we observed in the engineered iCCA cell lines (Fig. 3).

To obtain the target genes affected both by p63 knockdown and p63 overexpression, we picked out the 1196 overlapped DEG detected both in RBE and CCLP1 groups (Fig. 4C) and performed Gene Ontology (GO) and Kyoto Encyclopedia of Genes and Genomics (KEGG) analysis. These overlapped DEG showed significant enrichment in biological process correlated with cell adhesion, cytoskeleton organization and extracellular matrix (ECM) organization. PI3K/Akt, MAPK, Wnt, Notch and NF-κB signaling pathways were also significantly enriched (Fig. 4D; Figure S7). We displayed the expression pattern of DEG involved in cell proliferation, migration and invasion in chord diagrams (Fig. 4E) and found that majority of these DEG were downregulated in CCLP1 group while upregulated in RBE group. In particular, genes encoding intermediate filament protein such as KRT3, KRT5, KRT15 and KRT17 displayed decreased expression in p63 knockdown group and increased expression in p63 overexpression group; integrins like ITGA3, ITGA2 and ITGB4 showed concordant expression with p63; cell adhesion molecules COL17A1, FAT2 and NECTIN1 were downregulated while knocked down p63 and upregulated when overexpressed p63; MMP14, a critical proteolytic enzyme degrading ECM and promoting cell migration and invasion, was significantly increased as p63 overexpressed and decreased when p63 knocked down (Fig. 4E). Results of enrichment analysis were summarized in the Supplementary Data (Tables S4–S6).

We further applied the 1196 overlapped DEG to protein-protein interaction (PPI) network analysis to determine the hub genes correlated with p63 (Fig. 4F). Besides intermediate filament protein and cell adhesion molecules mentioned above, the following hub genes were also picked out: PTEN, the negative modulator which independently inhibit PI3K pathway and thus restrains cell migration and invasion [41]; S100A2, which enhances PI3K/AKT activation to promote tumour cell proliferation and invasion [42]; JAG1, important molecule that triggers Notch signaling which can crosstalk between PI3K/AKT, NF-κB or integrin signaling pathways to facilitate tumour metastasis [43]; SFN, which has been reported to be associated with the metastatic properties of CCA cells and leads to worse patient outcomes [44]; and IRF6, which regulates cell adhesion and necessary for cell migration in epithelial cells [45]. Heatmap was drawn to display the expression pattern of these hub genes in engineered iCCA cell lines, among which COL17A1, FAT2, IRF6, JAG1, KRT15, KRT17, NECTIN1, S100A2, SFN showed consistent expression with p63 (Fig. 4G). We then performed correlation analysis of p63 and these target genes at the RNA level by using transcriptomic profiles from the NODE dataset (OEP001105), and found COL17A1, KRT15, KRT17, S100A2, SFN to be significant positively correlated with p63 (Fig. 4H), survival analysis further revealed their correlation with poor survival in iCCA (Fig. 4I). Among these target genes, KRT17 expression was validated by qRT-PCR and obtained results consistent with RNA-seq (Figure S8).

These findings suggest that p63 might induce cell migration and invasion to enhance cancer progression in iCCA via direct regulation of genes involved in cytoskeleton, cell adhesion, or PI3K pathways.

### Chromatin landscape alteration by abnormal p63 expression

**Fig. 5:**
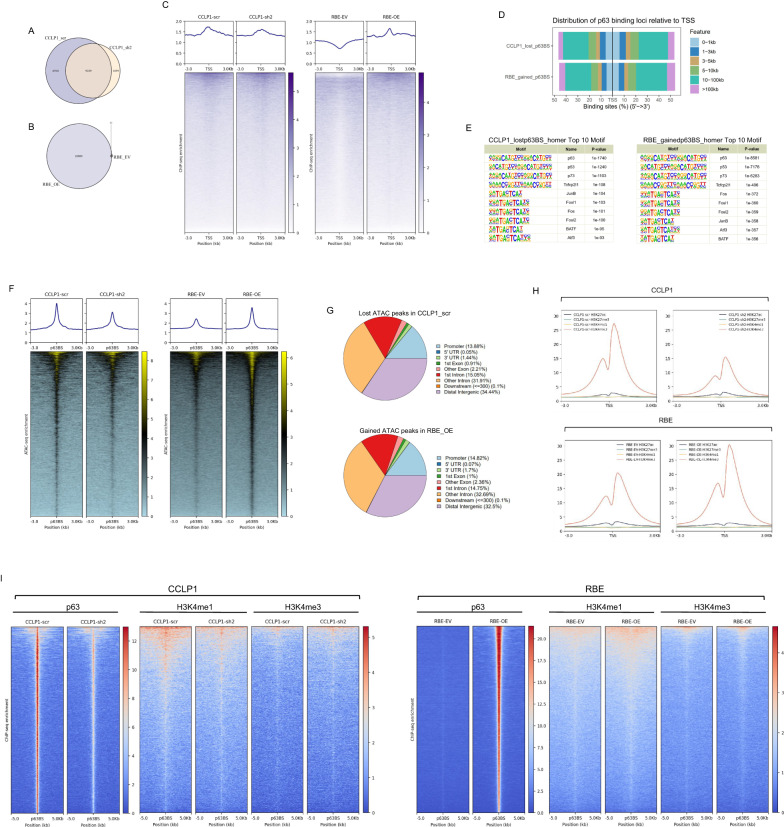
p63 binding remodels chromatin accessibility at its target sites and alters the chromatin landscape. **A** Venn diagram comparing identified p63 ChIP-seq peaks in CCLP1_scr and CCLP1_sh2, **B** Venn diagram comparing identified p63 ChIP-seq peaks in RBE_OE (RBE_over) and RBE_EV (RBE_vector). **C** Metaplot and heatmap of p63 ChIP-seq reads 3 kb upstream and 3 kb downstream of each TSS in CCLP1_scr and CCLP1_sh2, RBE_OE and RBE_EV, respectively. **D** Barplot showing the distance of p63 peak summits from the TSS in CCLP1_lost_p63BS (CCLP1_scr vs CCLP1_sh2) and RBE_gained_p63BS (RBE_OE vs RBE_EV). **E** Top enrichment identified by HOMER known motif analysis on 500-bp regions centred on p63 peak summits in CCLP1_lost_p63BS and RBE_gained_p63BS respectively. **F** Metaplot and heatmap displaying the ATAC-seq signals from −3 kb to 3 kb surrounding the lost p63 peak summits (CCLP1_scr vs CCLP1_sh2) in CCLP1_scr and CCLP1_sh2, and the gained p63 peak summits (RBE_OE vs RBE_EV) in RBE_EV and RBE_OE. **G** Pie chart showing the genome-wide distribution of lost ATAC-seq peaks (CCLP1_scr vs CCLP1_sh2) in CCLP1_scr, and gained ATAC-seq peaks (RBE_OE vs RBE_EV) in RBE_OE. **H** Metaplot displaying the ChIP-seq signal of histone marks H3K27ac, H3K27me3, H3K4me1 and H3K4me3, 3 kb flanking the TSS in CCLP1_scr, CCLP1_sh2, RBE_EV, and RBE_OE, respectively. **I** Metaplot and heatmap displaying the ChIP-seq signal of p63, H3K4me1 and H3K4me3; data were plotted for 5 kb flanking the lost p63 peak summits (CCLP1_scr vs CCLP1_sh2) > 10 kb away from TSS in CCLP1_scr and CCLP1_sh2, and the gained p63 peak summits (RBE_OE vs RBE_EV) > 10 kb away from TSS in RBE_EV and RBE_OE, respectively.

p63 ChIP-seq was performed to detect its binding in iCCA cell lines. As expected, we obtained more p63-bound sites in CCLP1_scr than in CCLP1_sh2 and obtained tens of thousands of binding sites in RBE_over but almost none in RBE_vector (Fig. 5A, B). Analysis of the distribution of p63 binding in the genome revealed that only a small fraction of p63 binding occurred around the transcription start site (TSS), while the majority occurred in regions distal to the TSS (Fig. 5C). We next selected the lost p63 binding sites while knocking down p63 and the gained binding sites after p63 overexpression and found that most of these sites were also located distant from the TSS (Fig. 5D). Therefore, we hypothesized that p63 might regulate target gene expression via long-distance interactions. Motif analysis was performed on regions around these lost/gained p63 binding sites and revealed enrichment of the p53 family, including p53, p63, and p73; the FOS family, which regulates cell proliferation; and BATF, which has recently been shown to be a key regulator in modulating chromatin architecture [46] (Fig. 5E).

ATAC-seq was performed to determine the effect of p63 on chromatin structure. At the TSS, chromatin accessibility did not show any obvious changes (Figure S9A). We further analyzed the distribution of ATAC-seq peaks in the genome between different groups (CCLP1_sh vs. CCLP1_scr and RBE_over vs. RBE_vector) and found that those peaks were mainly located in the promoter, introns, and distal intergenic regions, but did not show obvious differences (Figure S9B). Next, we examined the ATAC signal at p63 gained/lost binding sites. The results showed that the target site accessibility obviously decreased or increased when p63 binding was lost or gained, respectively (Fig. 5F), indicating the capability of p63 to remodel the chromatin structure at its target sites after binding. We also analysed the lost/gained ATAC-seq peaks in CCLP1 and RBE and found that these sites exhibited a large percentage of non-coding regions, such as introns and distal intergenic regions (Fig. 5G), indicating that p63 plays a role in reshaping the chromatin structure at these distal regulatory regions to establish a more accessible environment.

H3K27ac, H3K27me3, H3K4me1, and H3K4me3 ChIP-seq were conducted to detect the chromatin state during changes in p63 expression. We first analyzed the chromatin state around the TSS, and found histone modifications such as H3K27ac, H3K27me3, and H3K4me1 did not show evident change, but highly enriched H3K4me3 signals were detected in p63-high-expressing cells (CCLP1_scr and RBE_over) compared to p63-low/no-expressing cells (CCLP1_sh and RBE_vector) (Fig. 5H). Previous studies have found that H3K4me3 serves as a recognition platform to recruit chromatin remodelling factors, such as CHD1, to establish transcription favouring an open chromatin state [47]. Moreover, H3K4me3 is a predominant feature of active promoters, but it can also be detected at relatively low levels in active enhancers [48]. High levels of H3K4me1 relative to H3K4me3 can distinguish enhancers from promoters [49]. In our results, p63 binding sites with high H3K4me3 showed barely detectable H3K4me1, while regions with high H3K4me1 had relatively low H3K4me3 signals; since the majority of p63 binding occurred in regions distant from the TSS, we supposed that this p63 binding might correlate with enhancer regulation. Therefore, we further classified the p63 binding sites into the distal sites (>10 kb away from the TSS) and proximal sites (<1 kb away from the TSS) and analysed the H3K4me1 and H3K4me3 signals around the distal sites. As expected, the distal sites showed higher H3K4me1 to H3K4me3 ratio (Fig. 5I) than sites around the TSS (Fig. 5H). Our results indicate that even though p63 binding mostly occurred at enhancer regions, the chromatin state near the TSS can also be affected; therefore, we hypothesize that long-distance chromatin interactions might exist between the promoter and p63-bound enhancer.

### Interaction of p63 with chromatin structural protein YY1

**Fig. 6:**
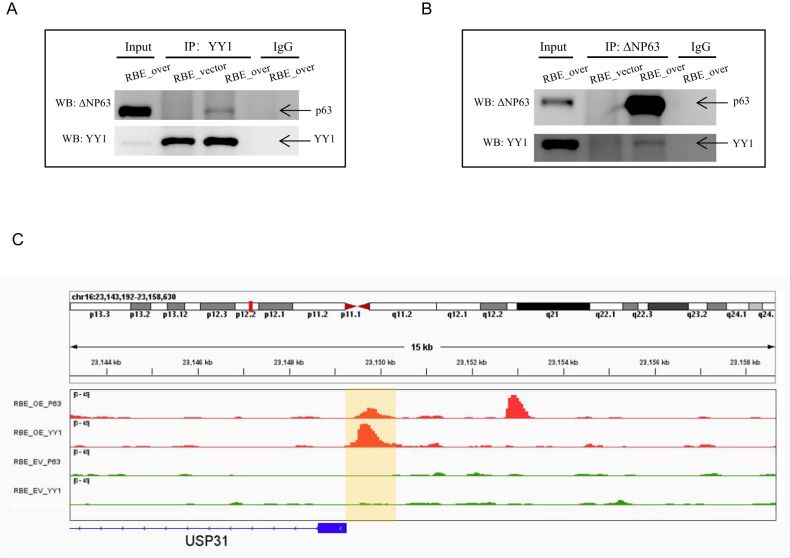
p63 interaction with YY1 protein. **A**, **B** Co-immunoprecipitation (Co-IP) assays showing the interaction between p63 and YY1. Protein extracts (input) were immunoprecipitated with anti-YY1 **A** or anti-ΔNp63 **B** antibodies and resolved using SDS-PAGE. Protein–protein interactions were immunoblotted using anti-ΔNp63 **A** and anti-YY1 antibodies **B**. **C** Integrative Genomics Viewer (IGV) image highlighting the p63 binding site overlap with the YY1 binding site, which was obtained from p63 ChIP-seq and YY1 ChIP-seq data.

The higher-order spatial organization of chromatin plays a vital role in gene regulation, and CTCF is a known architectural protein capable of establishing chromatin loops to connect regulatory elements to their target genes. A recent study has indicated that YY1 also functions as a structural regulator of enhancer–promoter loops [50]. However, CTCF is usually enriched at the TAD and rarely involved in enhancer–promoter loops, whereas YY1 binding occurs in the promoters and enhancers of human genes to establish distal chromatin interactions [50]. Studies have already indicated cooperation between p63 and CTCF in modulating chromatin architecture [51]. Therefore, we speculated whether YY1 also plays a role in p63-regulated enhancer–promoter interactions. It has already been shown that YY1 binds to the p53 protein [52], and p63 is a conserved homologue of p53; therefore, we investigated whether p63 can also interact with YY1. We conducted co-immunoprecipitation (Co-IP) experiments on the RBE_vector vs. RBE_over groups, and found that p63 and YY1 can bind to each other (Fig. 6A, B). Researchers have already reported that YY1 selectively binds to a subset of p53 target sites [53]. To test whether YY1 also binds p63 target sites, we further conducted YY1 ChIP-seq on the RBE group (RBE_vector vs. RBE_over). However, the YY1 level in RBE was very low; we only captured hundreds of YY1 targets and found that a small proportion of YY1 binding sites overlapped with p63 binding sites (Fig. 6C). Although we observed an interaction between p63 and YY1, their cooperation in vivo to regulate target genes requires further investigation.

## Discussion

In this study, we found that abnormal p63 expression in iCCA plays a role in tumour progression and is correlated with poor prognosis. Our results indicate that p63 not only affects cell proliferation and migration capabilities, but also remodels the chromatin landscape and regulates a set of oncogenes and cellular development-related genes. These findings shed light on the importance of p63 in the epigenetic modulation and genetic regulation of iCCA development.

The p63 gene has been shown to act either as a tumour suppressor gene or oncogene [54, 55], and early studies on p63 in tumours were confusing and contradictory owing to the various isoform types of p63 in humans. Depending on the presence of an N-terminal transactivation domain, p63 can be divided into either TAp63 or ΔNp63 isoforms, and alternative C-terminal splicing further generates different splice variants, α, β, and γ. TA isoforms of the p63 gene are regarded to preserve tumour-suppressive functions similar to those of p53, such as the induction of apoptosis, cell cycle arrest, and senescence [56], while ΔN isoforms of p63 act more like oncogenes, which can antagonize p53 [57]. ΔNp63α is the predominant isoform in epithelia-originated cells, and is found to be overexpressed in various tumours [58]. p63 expression was not observed in normal biliary epithelia, and p63 was not detected in normal intrahepatic epithelial cells [59]. However, p63 protein expression in iCCA has been reported in several studies [60, 61]. Therefore, we analysed the RNA-seq data from the TCGA database and detected p63 RNA expression in iCCA, although in most cases it was extremely low. The expression level of p63 was significantly correlated with clinical outcomes in iCCA; p63-high-expressing patients had lower survival rates than their p63-low-expressing counterparts. A recent study proposed that ΔNp63α facilitated biliary oncogenic transformation via ectopically expressing ΔNp63α in the bile duct epithelium of transgenic mice [28]. These findings suggest the participation of p63 in the development of iCCA.

We employed multiple iCCA cell lines to test p63 expression, but only found relatively high p63 expression in one of them, whereas the expression levels in the other cell lines were limited and not significantly different from those detected in normal intrahepatic bile duct epithelial cells. Our results were different from those of a previous study that detected diverse p63 expression in CCA tumour samples [61], but were consistent with a more recent study that observed p63 in a small fraction of CCA samples [26]. Moreover, our study analysed the expression of different p63 isoforms in iCCA not only at the protein level but also at the RNA level, whereas previous studies tested p63 proteins in CCA and did not distinguish different isoforms. As ectopic expression of ΔNp63α in mice bile duct epithelial cells facilitated biliary oncogenic transformation [28], we assumed that the abnormal expression of ΔNp63α in intrahepatic cholangiocytes was associated with iCCA tumour progression. Therefore, in this study, we investigated the role of the ΔNp63α isoform in iCCA.

p63 knockdown in CCLP1 cells displaying high p63 expression resulted in impaired cell growth, and overexpression of ∆Np63α in RBE cells, with barely detectable p63 levels, resulted in significantly increased cell proliferation and migration. Our RNA-seq analysis returned significantly altered genes involved in cytoskeleton organization, cell adhesion and ECM organization, correlated with cell proliferation, migration and invasion as revealed by functional enrichment analysis, these results are in concordance with the phenotype we observed in iCCA cell lines. COL17A1, KRT15, KRT17, S100A2, SFN, which have been shown to assume oncogenic roles in various cancers, were among the highly changed genes and positively correlated with p63 expression. These findings indicated an oncogenic role of ∆Np63α in iCCA via modulating mobility and invasiveness of cells.

In contrast to the classical transcription factors, p63 has been shown to regulate the chromatin landscape [51]. Scientists demonstrated the cooperation between p63 and the chromatin remodelling complex BAF to maintain open chromatin [62] and confirmed the importance of p63 in the establishment of epithelial enhancers [63]. Our recent study also confirmed the pioneering capability of p63 to bind inaccessible chromatin [12]. Based on these findings, we proposed that p63 regulates downstream target genes by reshaping the chromatin landscape in iCCA. We obtained p63-bound sites in different engineered iCCA cell lines through ChIP-seq and then performed ATAC-seq to analyse the chromatin accessibility changes at the lost and gained p63-bound sites in CCLP1 and RBE, respectively. As expected, p63-bound sites had increased accessibility compared to unbound sites, indicating the role of p63 in chromatin remodelling in iCCA. Moreover, even though the majority of p63 binding occurred at substantial distances from the TSS, it significantly increased the H3K4me3 signal near the TSS, which is a known marker for active promoters, indicating that p63 might establish long-distance enhancer–promoter interactions to activate target genes. Therefore, we further divided p63-bound sites into distal (>10 kb away from TSS) and proximal (<1 kb away from TSS) groups, and found that the distal group showed an enriched H3K4me1 to H3K4me3 ratio, which represents the enhancer, while the proximal group only displayed an enriched H3K4me3 signal, which is a mark for the promoter. In addition, the p63-bound sites showed increased H3K4me1 or H3K4me3 signals in both the distal and proximal groups, indicating that p63 reshaped its bound regions into a more active chromatin state.

Previous studies have revealed that CTCF and YY1 are enriched around p63 binding sites [51, 57], both of which function as chromatin organizers [50, 64], indicating that they may be associated with p63 to modulate chromatin architecture. Cooperation between p63 and CTCF has been reported [50]; however, CTCF is usually enriched at the boundary of topologically associated domains, and is rarely involved in the formation of enhancer–promoter loops [64]. In contrast, YY1 is involved in establishing the enhancer–promoter DNA loop [50]. p53 has been reported to bind YY1 [52], and p63 is a homologue of p53. Therefore, we investigated whether p63 can interact with YY1. We performed co-IP experiments and observed an interaction between p63 and YY1 in iCCA cells. However, the YY1 ChIP-seq assay did not capture enough YY1-bound sites, which might be due to the low YY1 expression levels in the RBE cells, suggesting that p63-YY1 cooperation might not be the key factor for long-distance gene regulation in RBE cells. Further investigation is required to determine how p63 cooperates with YY1 to regulate downstream target genes.

In summary, we explored the role of p63 in iCCA and identified its important role in iCCA cell proliferation and migration, the modulation of chromatin state, and the regulation of gene expression. Overall, this study provides novel insights into the regulatory role of p63 in iCCA and offers new ideas for exploring the complexity of iCCA.

### Supplementary information

Table.S1

Table.S2

Table.S3

Table.S4

Table.S5

Table.S6

Fig.S1

Fig.S2

Fig.S3

Fig.S4

Fig.S5

Fig.S6

Fig.S7

Fig.S8

Fig.S9

Full length uncropped original western blots

## Data Availability

The datasets generated during this study are available at NCBI SRA: PRJNA966810. Additional data related to this paper may be requested from the authors.
