# Supplementary material for: ΔNp63α facilitates proliferation and migration, and modulates the chromatin landscape in intrahepatic cholangiocarcinoma cells
Source: Cell Death Dis. 2023 Nov 27;14(11):777. doi: 10.1038/s41419-023-06309-7 (PMC10682000; doi:10.1038/s41419-023-06309-7)
Supplement: Supplementary file 8 — Fig.S2 [file 41419_2023_6309_MOESM8_ESM.pdf]

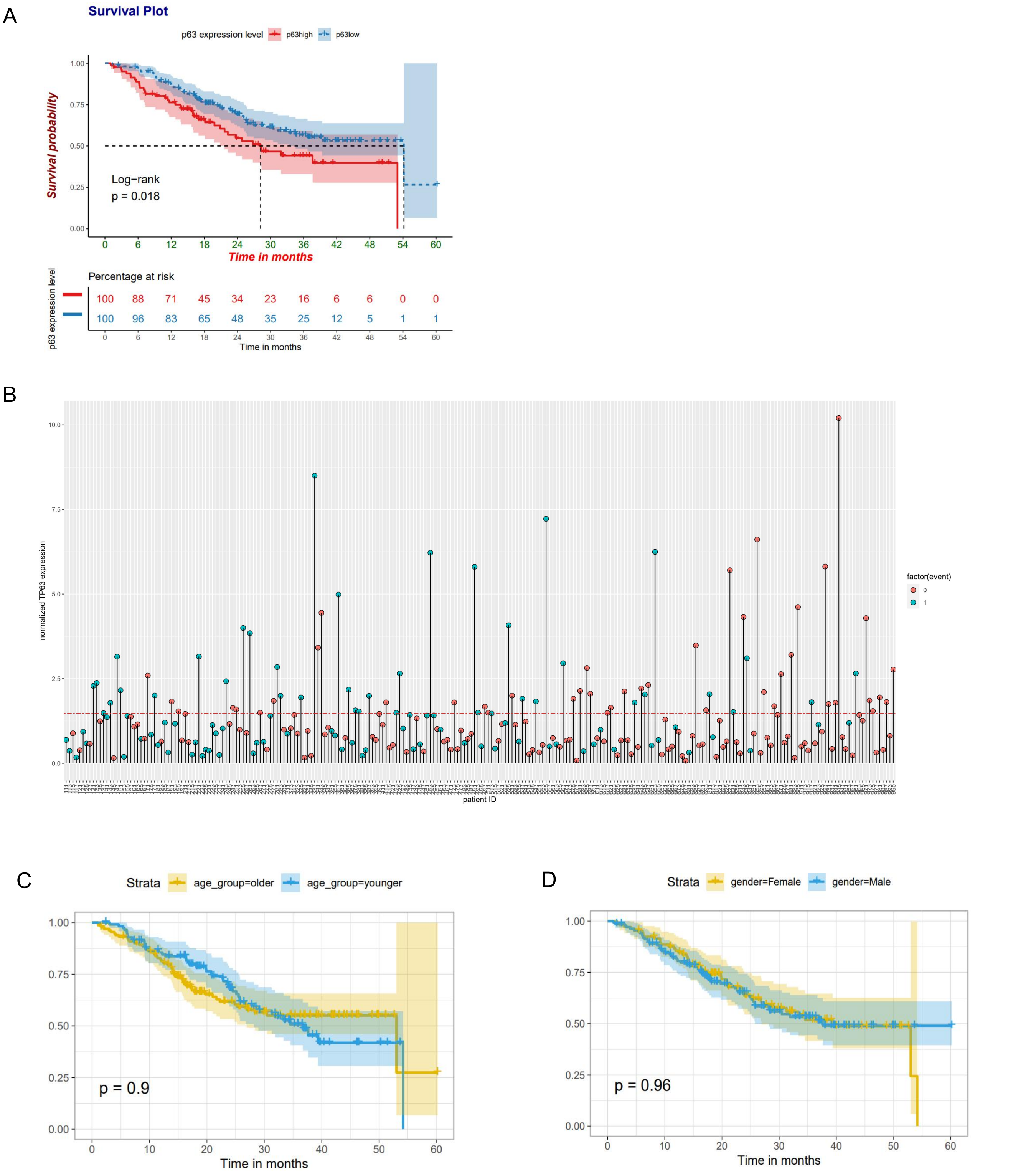

Fig. S2: Dataset from the NODE database (OEP001105, https://www.biosino.org/ node/project/detail/OEP001105) was used in this analysis. (A) Kaplan–Meier analysis comparing the survival of iCCA patients with low p63 expression to patients with high p63 expression using the mean value as the threshold. P-value was calculated using the log-rank test. (B) p63 expression in each individual iCCA patient from the NODE database (OEP001105) is displayed with clinical outcomes. Status indicator 0=alive, 1=dead. (C) Kaplan–Meier analysis comparing survival of older iCCA patients to younger iCCA patients using the mean value of age as the threshold. P-value was calculated using the log-rank test. (D) Kaplan–Meier analysis comparing survival of female iCCA patients with male patients. P-value was calculated using the log-rank test.
