# Supplementary material for: ΔNp63α facilitates proliferation and migration, and modulates the chromatin landscape in intrahepatic cholangiocarcinoma cells
Source: Cell Death Dis. 2023 Nov 27;14(11):777. doi: 10.1038/s41419-023-06309-7 (PMC10682000; doi:10.1038/s41419-023-06309-7)
Supplement: Supplementary file 9 — Fig.S3 [file 41419_2023_6309_MOESM9_ESM.pdf]

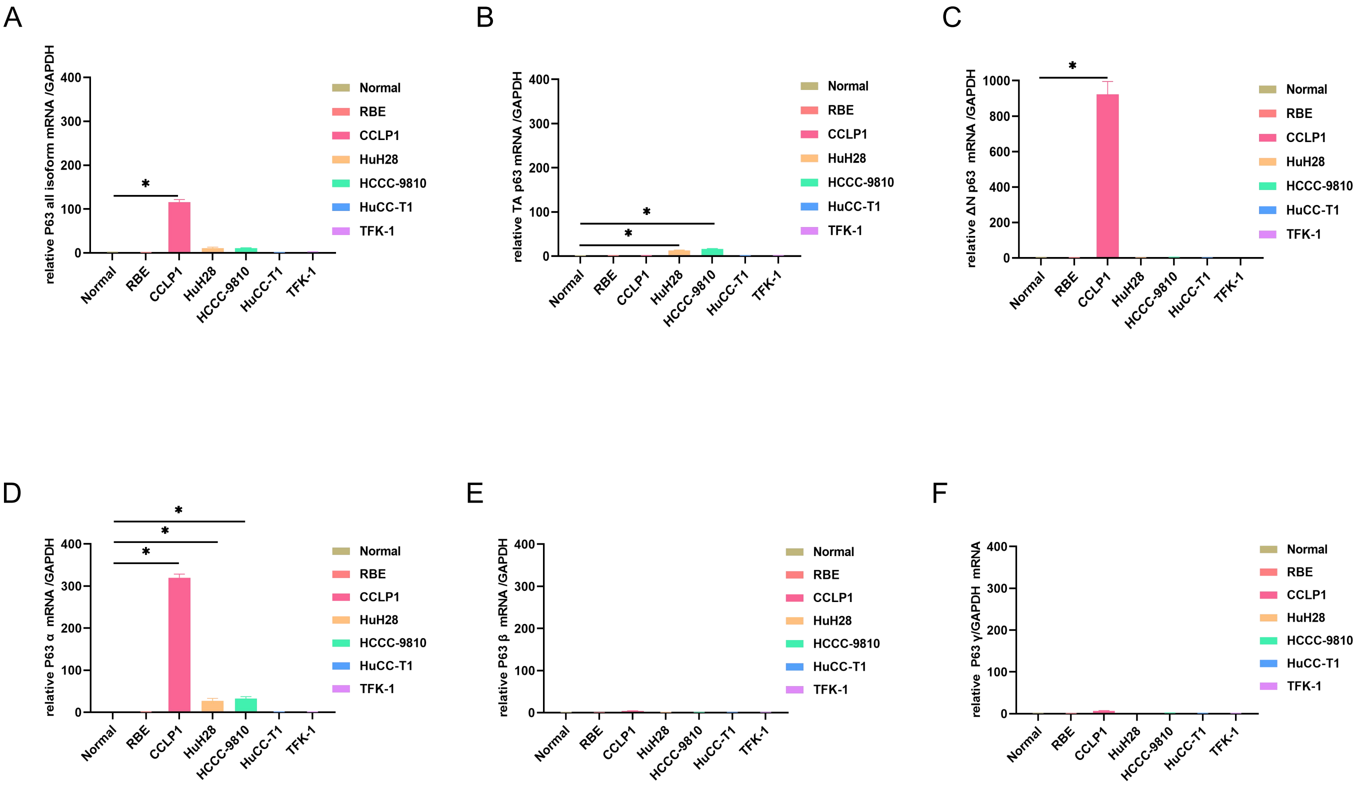

Fig. S3: qRT-PCR detected the expression of all isoforms (A) and the TA isoform (B), ΔN isoform (C), α isoform (D), β isoform (E), and γ isoform (F). Differences were considered significant at $p < 0.01$.
