# Supplementary material for: ΔNp63α facilitates proliferation and migration, and modulates the chromatin landscape in intrahepatic cholangiocarcinoma cells
Source: Cell Death Dis. 2023 Nov 27;14(11):777. doi: 10.1038/s41419-023-06309-7 (PMC10682000; doi:10.1038/s41419-023-06309-7)
Supplement: Supplementary file 10 — Fig.S4 [file 41419_2023_6309_MOESM10_ESM.pdf]

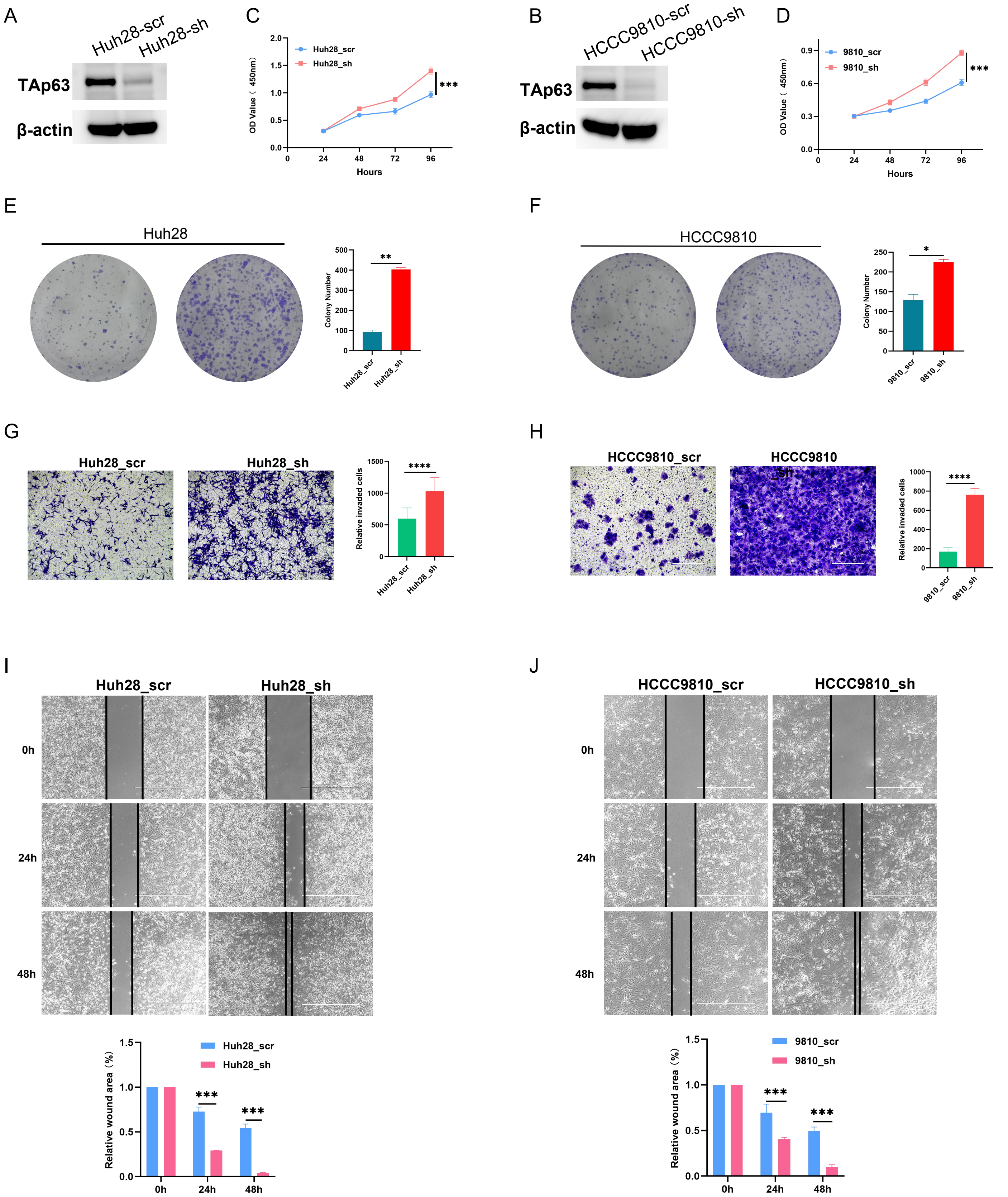

Fig. S4: (A) Huh28 cells were transfected with shRNA-expressing plasmid against the p63 gene to knockdown TAp63 and generate Huh28_sh; scrambled shRNA was used as a negative control to generate Huh28_scr. (B) HCCC9810 cells were transfected with shRNA-expressing plasmids against the p63 gene to knockdown TAp63 and generate HCCC9810_sh; scrambled shRNA was used as a negative control to generate HCCC9810_scr. (C) CCK-8 test showed that TAp63 downregulation significantly promoted the proliferation of Huh28. (D) CCK-8 test showed that TAp63 downregulation significantly promoted the proliferation of HCCC9810 cells. (E) Colony formation assay of Huh28_scr and Huh28_sh with statistical results shown on the right. (F) Colony formation assay of HCCC9810_scr and HCCC9810_sh with statistical results shown on the right. (G) Transwell assay revealed that the migration ability of Huh28 was significantly increased when TAp63 was knocked down. (H) Transwell assay revealed that the migration ability of HCCC9810 was significantly increased when TAp63 was knocked down. Wound healing assay showed that p63 knockdown enhanced the migration of Huh28 cells (I) and HCCC9810 cells (J). Data shown are representative results of repeated experiments. P-values were calculated using t-tests; * indicates $p < 0.05$, ** indicates $p < 0.01$, *** indicates $p < 0.001$, and **** indicates $p < 0.0001$.
