# Supplementary material for: ΔNp63α facilitates proliferation and migration, and modulates the chromatin landscape in intrahepatic cholangiocarcinoma cells
Source: Cell Death Dis. 2023 Nov 27;14(11):777. doi: 10.1038/s41419-023-06309-7 (PMC10682000; doi:10.1038/s41419-023-06309-7)
Supplement: Supplementary file 11 — Fig.S5 [file 41419_2023_6309_MOESM11_ESM.pdf]

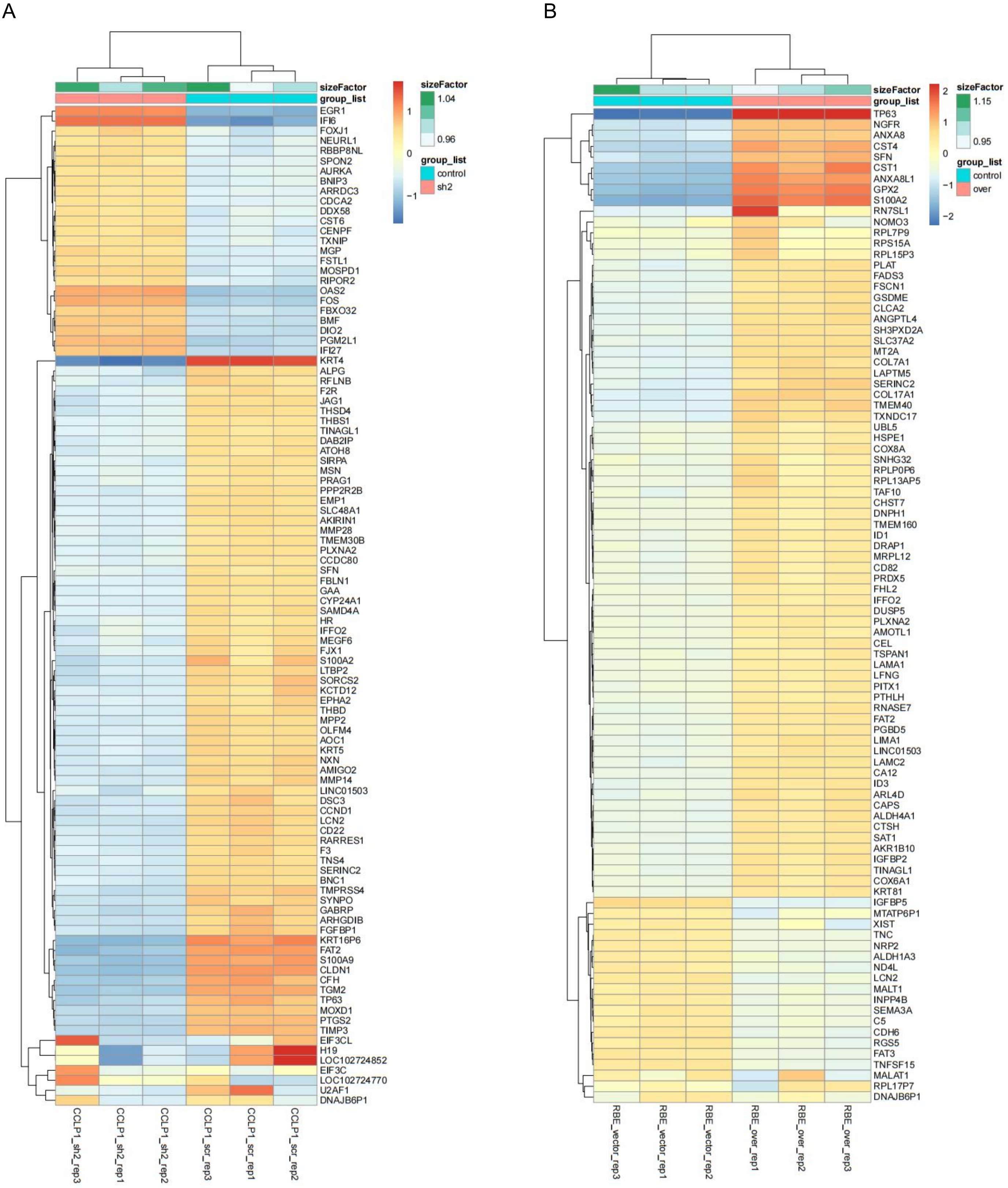


**Fig. S5:** Top 100 most variable genes obtained from RNA-seq in the CCLP1 group (A) and RBE group (B) to draw heatmaps.
