# Supplementary material for: ΔNp63α facilitates proliferation and migration, and modulates the chromatin landscape in intrahepatic cholangiocarcinoma cells
Source: Cell Death Dis. 2023 Nov 27;14(11):777. doi: 10.1038/s41419-023-06309-7 (PMC10682000; doi:10.1038/s41419-023-06309-7)
Supplement: Supplementary file 13 — Fig.S7 [file 41419_2023_6309_MOESM13_ESM.pdf]

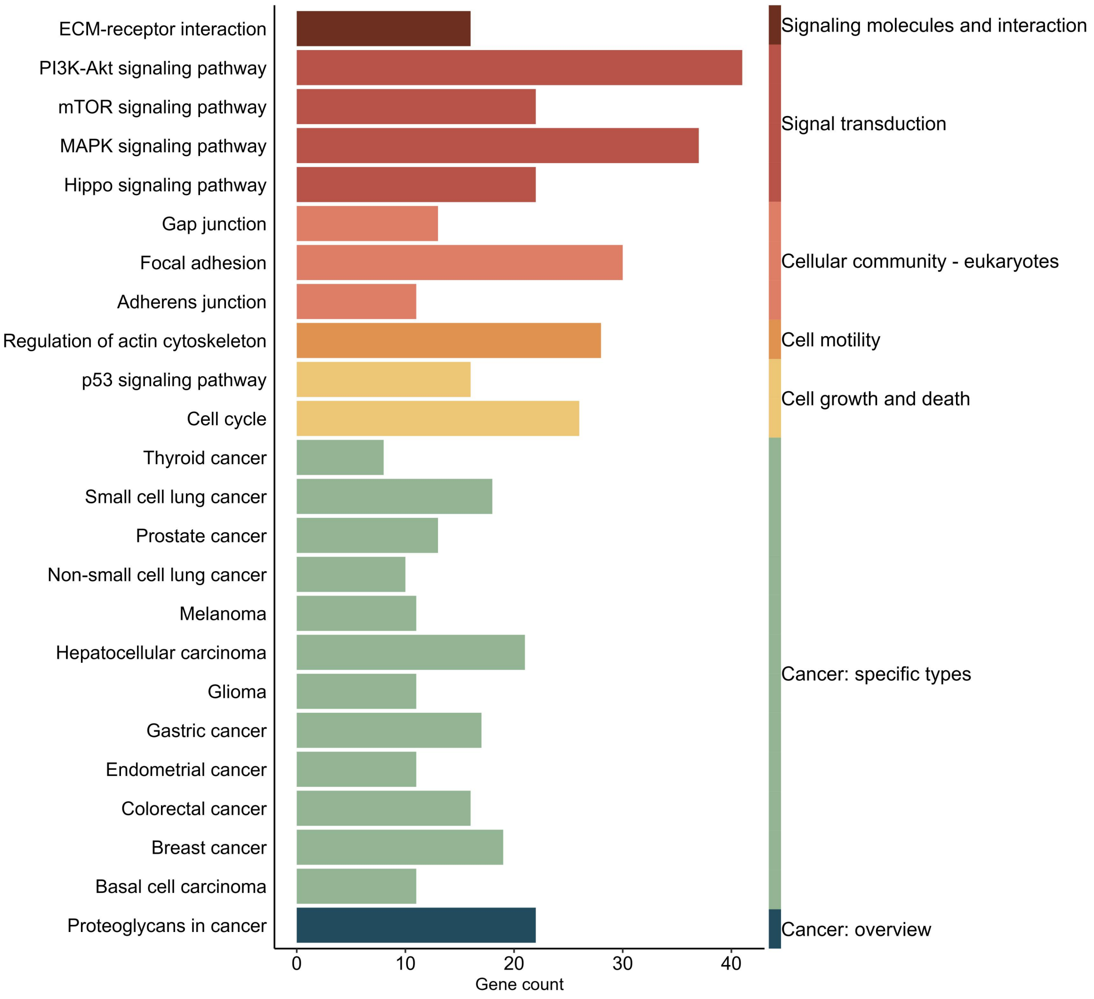

Fig. S7: KEGG analysis was performed on overlapped DEG, significantly enriched terms were displayed in the barplot with $p < 0.05$. Description of each term was written on the left, while correspond category was listed on the right.
