# Supplementary figures and images for: ΔNp63α facilitates proliferation and migration, and modulates the chromatin landscape in intrahepatic cholangiocarcinoma cells
Source: Cell Death Dis. 2023 Nov 27;14(11):777. doi: 10.1038/s41419-023-06309-7 (PMC10682000; doi:10.1038/s41419-023-06309-7)

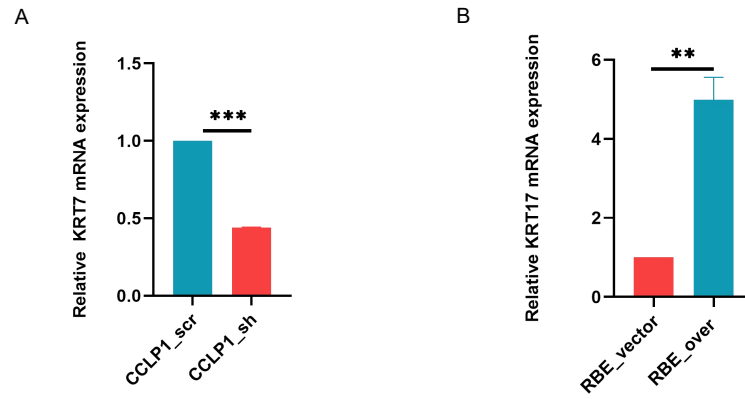

**Fig. S8:** KRT17 expression verified by qRT-PCR in the CCLP1 group (A) and RBE group (B).

Supplement: Supplementary file 14 — Fig.S8 [file 41419_2023_6309_MOESM14_ESM.pdf]

Fig2C

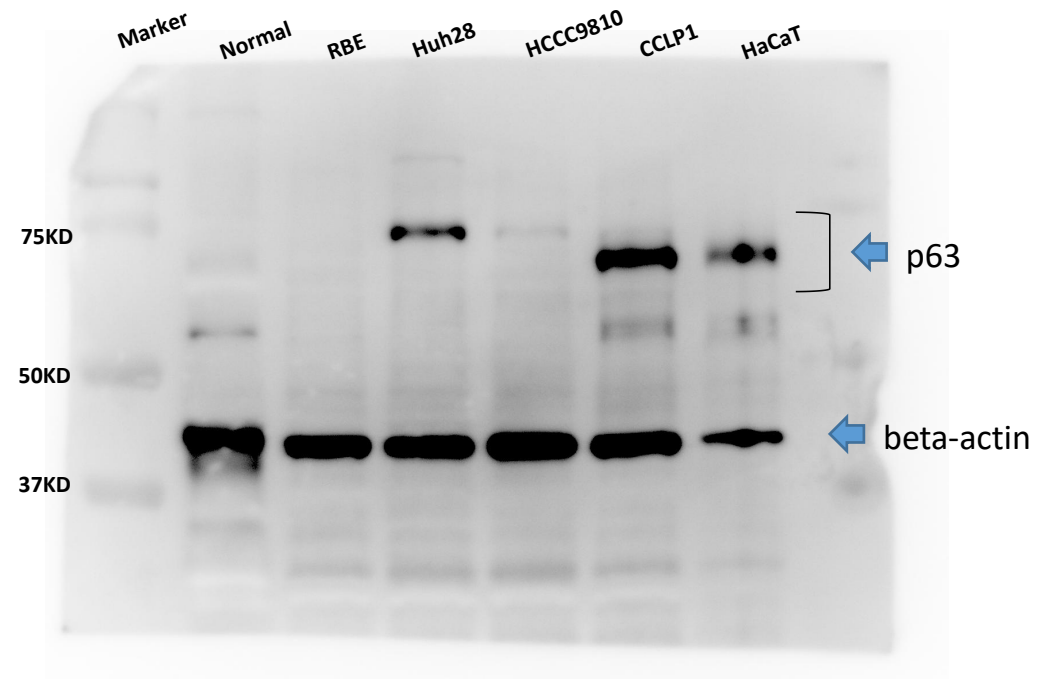

Fig3A,B

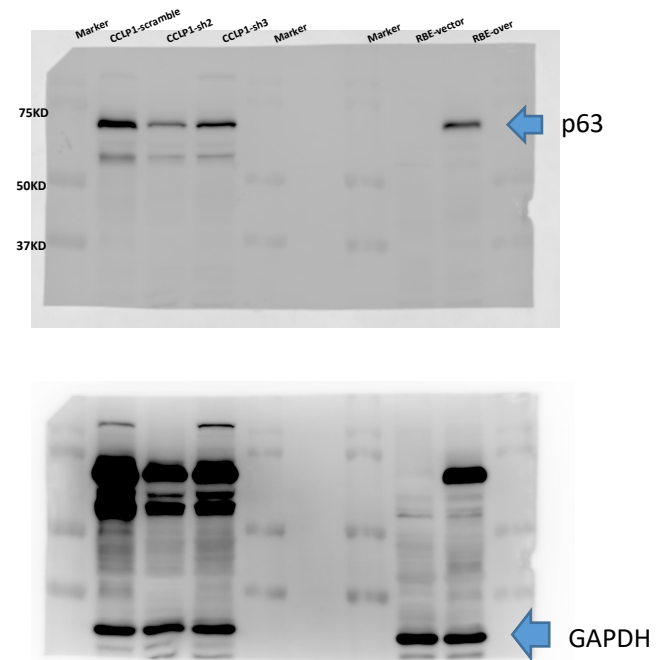

FigS4A,B

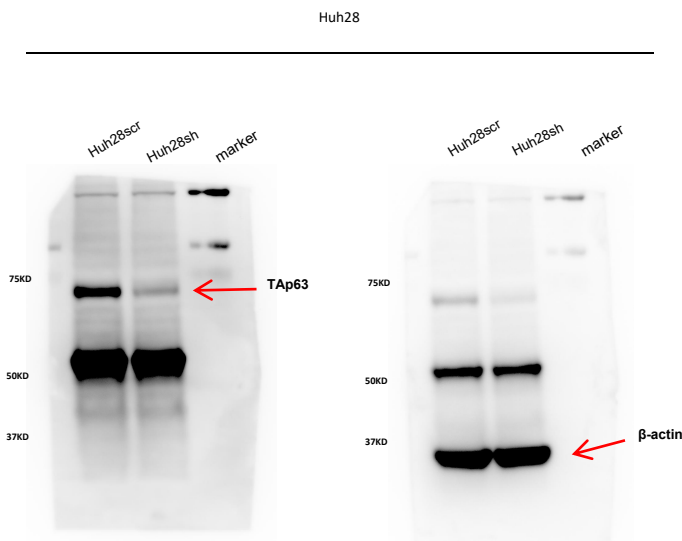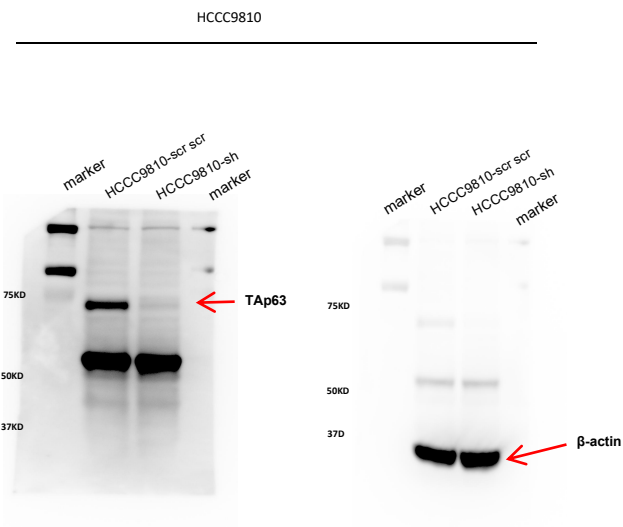

Fig6A,B

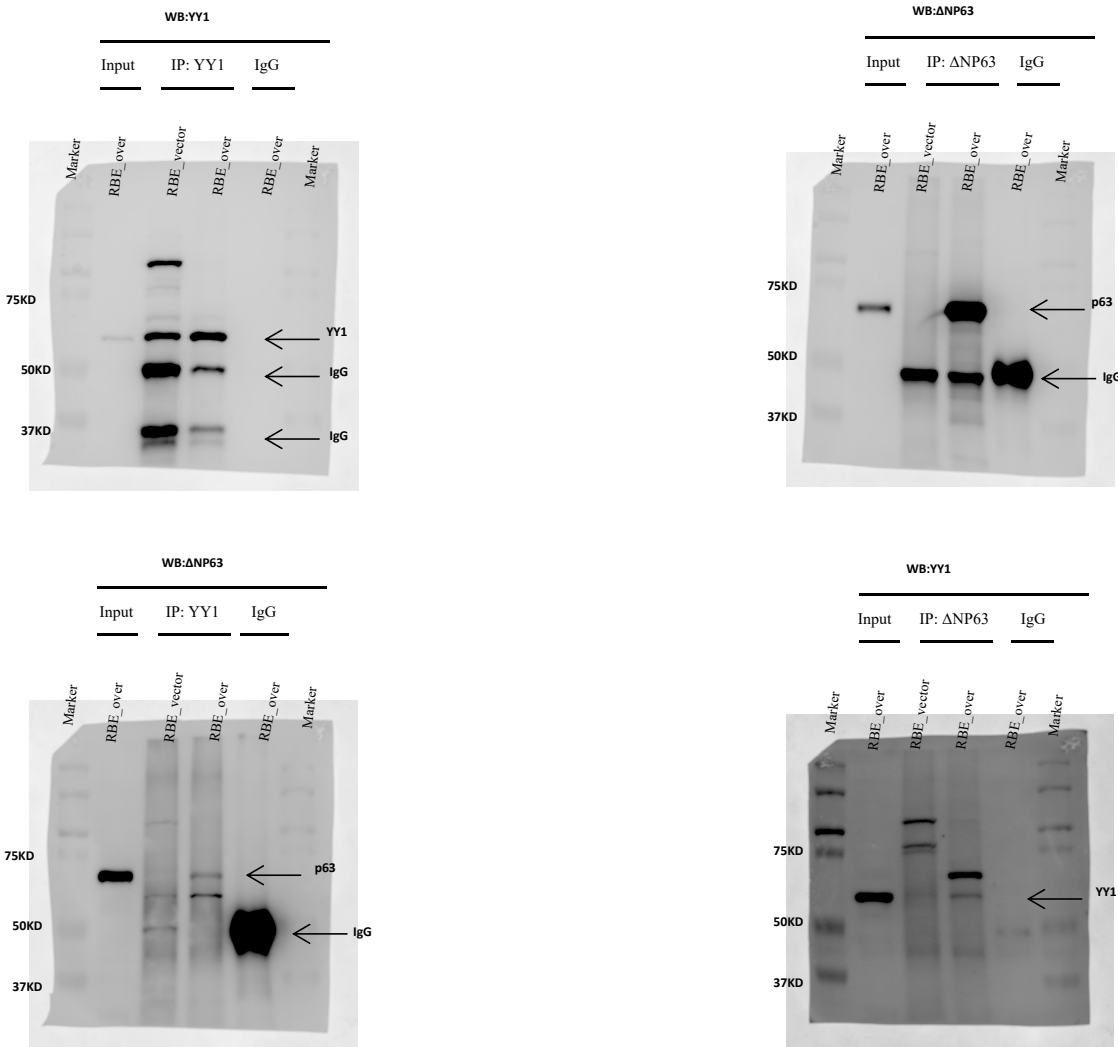

Supplement: Supplementary file 16 — Full length uncropped original western blots [file 41419_2023_6309_MOESM16_ESM.pdf]
