# Supplementary material for: ΔNp63α facilitates proliferation and migration, and modulates the chromatin landscape in intrahepatic cholangiocarcinoma cells
Source: Cell Death Dis. 2023 Nov 27;14(11):777. doi: 10.1038/s41419-023-06309-7 (PMC10682000; doi:10.1038/s41419-023-06309-7)
Supplement: Supplementary file 15 — Fig.S9 [file 41419_2023_6309_MOESM15_ESM.pdf]

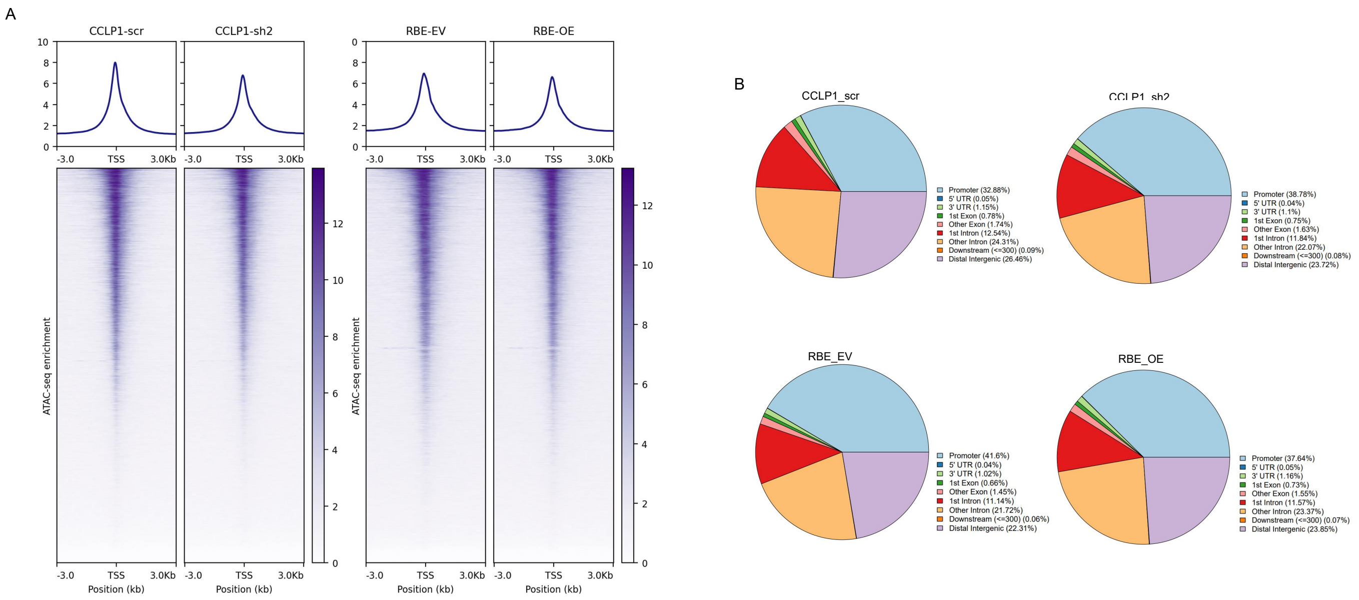

Fig. S9: (A) Metaplot and heatmap displaying the ATAC-seq signals from −3 kb to 3 kb surrounding the TSS in CCLP1_scr, CCLP1_sh2, RBE_EV, and RBE_OE, respectively. (B) Pie chart showing the genome-wide distribution of ATAC-seq peaks in CCLP1_scr, CCLP1_sh2, RBE_EV, and RBE_OE, respectively.
