# Supplementary material for: ΔNp63α facilitates proliferation and migration, and modulates the chromatin landscape in intrahepatic cholangiocarcinoma cells
Source: Cell Death Dis. 2023 Nov 27;14(11):777. doi: 10.1038/s41419-023-06309-7 (PMC10682000; doi:10.1038/s41419-023-06309-7)
Supplement: Supplementary file 7 — Fig.S1 [file 41419_2023_6309_MOESM7_ESM.pdf]

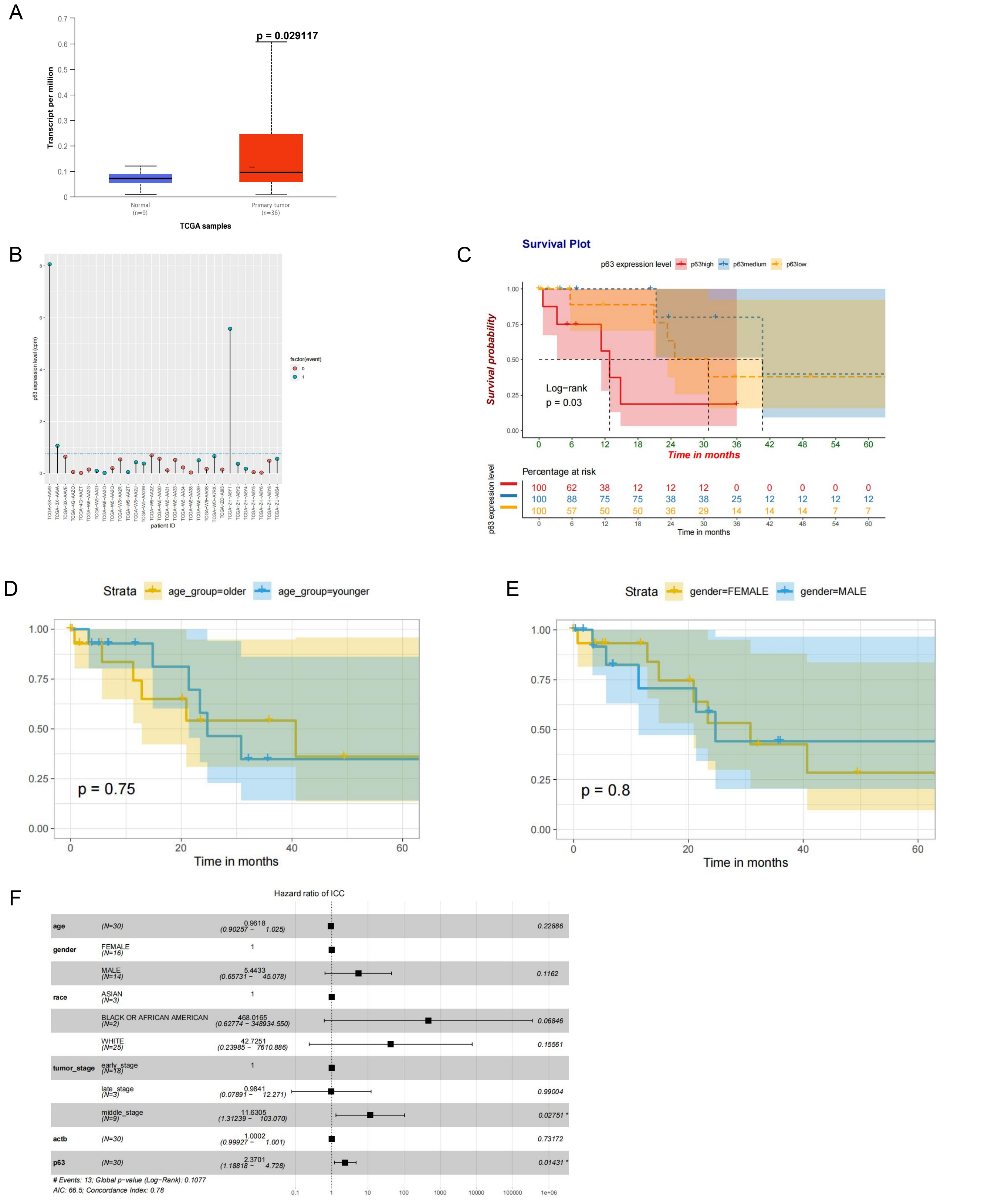

Fig. S1: (A) p63 expression in cholangiocarcinoma tumour and normal samples was visualized using UALCAN. (B) p63 expression in each individual iCCA patient from TCGA database is displayed with clinical outcomes. Status indicator 0=alive, 1=dead. (C) iCCA patients were classified into high, medium, and low p63 groups according to the upper, medium, and lower quartiles, respectively, of the p63 expression levels, and we applied Kaplan–Meier analysis to compare the survival of these three groups. (D) Kaplan–Meier analysis comparing survival of older iCCA patients to younger iCCA patients using the mean value of age as the threshold. P-value was calculated using the log-rank test. (E) Kaplan–Meier analysis comparing survival of female iCCA patients with male patients. P-value was calculated using the log-rank test. (F) Multivariate Cox regression analysis shows that p63 expression is associated with an increased hazard ratio (HR). Forest plot HR error bars represent 95% confidence interval, and the log-rank test was used to calculate the P-values.
