# Supplementary material for: ΔNp63α facilitates proliferation and migration, and modulates the chromatin landscape in intrahepatic cholangiocarcinoma cells
Source: Cell Death Dis. 2023 Nov 27;14(11):777. doi: 10.1038/s41419-023-06309-7 (PMC10682000; doi:10.1038/s41419-023-06309-7)
Supplement: Supplementary file 12 — Fig.S6 [file 41419_2023_6309_MOESM12_ESM.pdf]

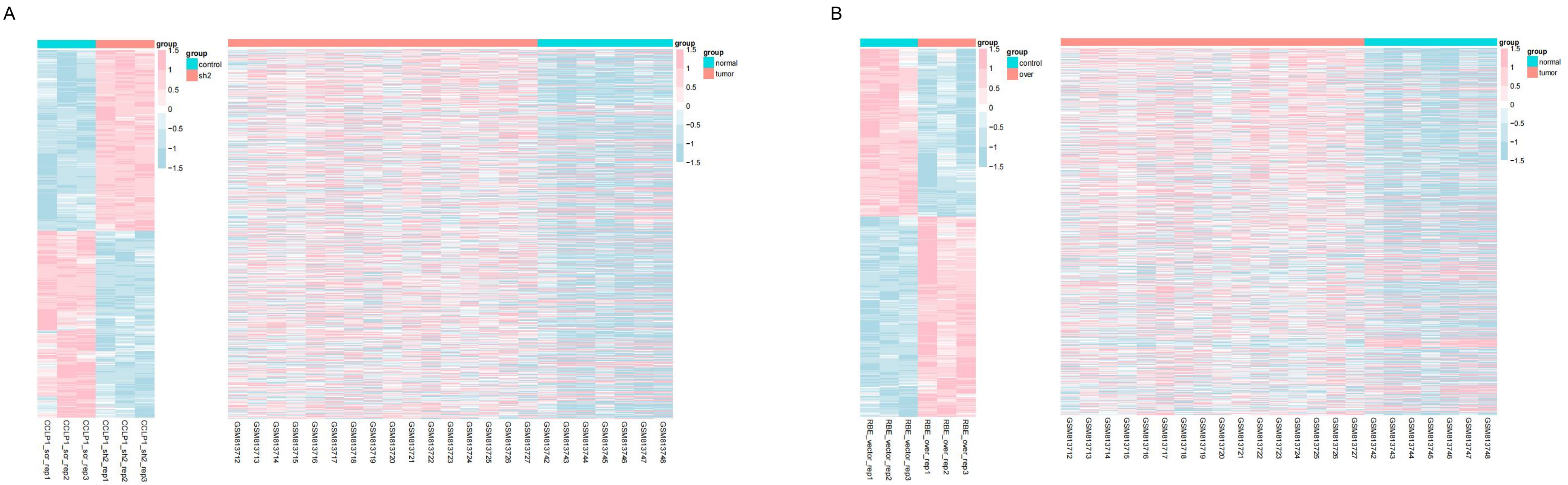

Fig. S6: (A) All differentially expressed genes (|log2FoldChange| > 0 and FDR < 0.05) were chosen. The 2611 downregulated and 2533 upregulated genes, as determined by RNA-seq from CCLP1, and the expression levels of these 5144 significantly altered genes were analysed in the transcriptomic profiles from GSE32879 (containing 16 ICC and 7 non-tumour tissues). (B) All differentially expressed genes (|log2FoldChange| > 0 and FDR < 0.05) were chosen. The 2233 downregulated and 2651 upregulated genes, as determined by RNA-seq from RBE, and the expression levels of these 4884 significantly altered genes were also analysed in the transcriptomic profiles from GSE32879.
